# Factors associated with compulsive sexual behavior in a national probability web-based aged 18-59 years sample from Japan: a cross-sectional survey

**DOI:** 10.1093/sexmed/qfaf091

**Published:** 2025-11-04

**Authors:** Yushun Okabe, Daisuke Ito

**Affiliations:** School of Allied Health Sciences, Kitasato University, Sagamihara, Kanagawa 252-0373, Japan; Research Fellow of Japan Society for the Promotion of Science, Chiyoda-ku, Tokyo 102-0083, Japan; Graduate School of Education, Hyogo University of Teacher Education, Kobe, Hyogo 653-0036, Japan

**Keywords:** compulsive sexual behavior disorder, sexual behavior, treatment-seeking behavior, depression, anxiety, ADHD

## Abstract

**Background:**

Compulsive sexual behavior disorder (CSBD) involves persistent, repetitive sexual behaviors that continue despite efforts to stop, leading to significant distress or impairment in personal, family, social, educational, and occupational functioning. Most compulsive sexual behavior (CSB) research focuses on Western countries, leaving its characteristics in Japan largely unexplored. Although treatment-seeking behavior for CSB is documented in Western contexts, there is a need to understand this behavior in Japan to improve mental health services.

**Aims:**

This cross-sectional study aimed to clarify association between CSB, sociodemographic factors, maximum sexual behavior duration, life satisfaction, negative consequences, psychological co-morbidities, and treatment-seeking behaviors in Japan.

**Methods:**

Participants were recruited from a national probability-based sample of adults aged 18-59 in Japan using a web-based questionnaire, with proportional representation of sex assigned at birth, age groups, and residential areas. A total of 1094 respondents (43.69% heterosexual cisgender men, *M*_age_ = 39.9 ± 11.6) were included.

**Outcomes:**

Participants completed the Compulsive Sexual Behavior Disorder Scale-19 (mean score = 24.35 ± 9.27), reporting sexual behavior frequency, time spent on sexual behaviors, life satisfaction, negative consequences related to sexual behaviors, treatment-seeking behaviors, and comorbid conditions such as depression (Patient Health Questionnaire-9), anxiety (Generalized Anxiety Disorder-7), and Adult Attention-deficit Hyperactivity Disorder (ADHD) (Adult Attention-deficit Hyperactivity Disorder Self-Report Scale).

**Results:**

Individuals at high risk of CSBD (*n* = 28; 2.56%), based on the CSBD-19 cut-off score, were more likely to be cisgender heterosexual men than women (chi-squared test). Compared to low-risk individuals, those at high risk spent more maximum duration on pornography and masturbation, lower life satisfaction in health and intimate relationships, experienced negative consequences in various life domains (eg, sleep, social activities, work, family life, finances, and physical health), and exhibited symptoms of depression, anxiety, and ADHD. Three individuals at high risk of CSBD reported seeking treatment.

**Clinical Implications:**

Individuals at high risk of CSBD experience negative consequences and psychological co-morbidities, making it critical to investigate contributing factors to provide effective treatment for CSB in Japan.

**Strengths and Limitations:**

This study contributes to limited research on CSB in non-Western cultures using a nationally probability-based sample in Japan. The measures lacked detail, so future studies should include more specific questions to evaluate treatment-seeking and other conditions. The small sample size limits the generalizability of the findings when comparing high- and low-risk CSBD groups.

**Conclusion:**

This study identified characteristics of individuals at high risk of CSBD in Japan, highlighting the need to develop a support system specific to CSB to ensure effective treatment.

## Introduction

Repetitive and intense sexual behaviors are increasingly recognized as clinically significant, prompting growing empirical research.^1^ The International Classification of Diseases, 11th Revision (ICD-11) classifies compulsive sexual behavior disorder (CSBD) as an impulse control disorder.^2^ CSBD is characterized by persistent inability to control repetitive sexual behaviors and urges. CSBD leads to significant distress and impairment across personal, family, social, educational, and occupational domains.

Research from non-Western contexts remains limited, highlighting the need for culturally diverse studies.^1^ However, compulsive sexual behavior (CSB) remains largely unexplored in non-Western societies.

In Japan, sex education remains underdeveloped and conservative, yet commercial sexual activities, such as pornography, are culturally accepted. However, the characteristics of individuals with high levels of CSB in Japan remain unclear. This study aimed to examine such characteristics of individuals at high risk of experiencing CSBD in Japan using a national probability-based sample in terms of the associations between CSB and sociodemographic factors, sexual behavior duration, life satisfaction, negative consequences, psychological co-morbidities, and treatment-seeking behaviors.

Specifically, we addressed the following question: (1) Are single, young men with low education, very high or very low income, full-time jobs, no children, living in a large city or alone, and sexual and gender minorities more likely to be at high risk of experiencing CSBD? Regarding problematic pornography use (PPU), tolerance (seeking more stimulating content), diversity of pornographic content, and binge-like behavior have been linked to CSB. We further hypothesized: (2) Do individuals at high risk of experiencing CSBD engage in more diverse and frequent sexual activities? (3) Do they report longer daily or session-based durations of sexual activity over the past year? (4) Do they experience lower quality of life (QOL) and reduced satisfaction in relationships and sexual life? Regarding the negative consequences on sexual life, individuals with high levels of CSB may seek more intense sexual stimulation, which might be linked to sexual risk behaviors, such as unprotected sex. We hypothesized: (5) Are they more likely to report sexually transmitted infections (STIs) or experience an abortion due to an unwanted pregnancy? (6) Are they more likely to experience adverse consequences (eg, work impairment, strained relationships, poor sleep)? (7) Do they more frequently report psychological symptoms? (8) To what extent do they seek treatment for their condition or for other disorders or conditions associated with out-of-control sexual behavior? This is particularly relevant as patterns of impaired control over sexual impulses, urges, or behaviors may manifest in individuals with mental health conditions such as bipolar disorder, those on medication for Parkinson’s disease, substance use problems, and individuals with dementia or brain injury. As a cross-sectional study, our findings reflect co-occurrence and cannot establish causality.

## Methods

### Participants and procedures

This study followed the Strengthening the Reporting of Observational Studies in Epidemiology (STROBE) statement for cross-sectional studies. An online survey through the survey company, targeting participants aged 18-59 years, was conducted in Japan from April 6 to 11, 2022 (http://www.cm-group.co.jp/). The final analysis, 1094 participants (*Mean* age = 39.9 ± 11.6 years) were included. Data were collected based on the proportionality of sex assigned at birth, age group, and residential areas in accordance with the 2020 Japanese Population Census statistics.

This study adhered to the principles of the 1964 Declaration of Helsinki (amended in 2013) and was approved by the ethical review board of the second authors’ institution on March 8, 2022 (approval number 2021-48). Informed consent was obtained from all participants. This study is part of a larger survey registered on the Open Science Framework (https://doi.org/10.17605/OSF.IO/29P5B), portions of which have been reported elsewhere.^3^

Participants were categorized into three groups for analysis: heterosexual cisgender men (*n* = 478; 43.69%), heterosexual cisgender women (*n* = 461; 42.14%), and sexual and gender minorities (*n* = 155; 14.17%). Details of this procedure are provided in the Supplementary Material.

### Measures

Detailed descriptions of the measures are in the Supplementary Material.

#### Sociodemographic data

Participants reported their age, sex assigned at birth, gender identity, sexual orientation, income, employment status, relationship status, whether or not have children, highest level of education, residence, and living situation.

#### Compulsive sexual behavior

The Compulsive Sexual Behavior Disorder Scale-19 (CSBD-19)^3^ assessed compulsive sexual behavior over the past 6 months. The total score ranges from 19 to 76 points, with higher scores indicating more severe CSB.

#### Sexual behaviors

Participants reported the frequency of sexual behaviors, including visiting sex establishments, telephone sex, and live streaming sex over the past 1 year, and the average time spent on sexual behaviors such as pornography use, masturbation, telephone sex, and cybersex during a single session over the past year. The longest duration spent on sexual behaviors in a single day over the past year was also reported.

*Life satisfaction* **.**

Five items from the World Health Organization (WHO) Quality of Life-BREF^4^ assessed health, daily physical activity, accessibility to health services, and self-satisfaction to gain insights into their overall health. Three additional items measured satisfaction with relationships at work or school, friendships, and family relationships. The Quality Marriage Index^5^ (QMI) was adapted to assess partner relationships by replacing the term “marriage” with “partner.” Additionally, 2 items from the International Index of Erectile Function were used to assess sexual life quality.^6^

*Negative consequences* **.**

To assess negative consequences in daily life, 5 questions modified from the Sheehan Disability Scale,^7^ were used. These included three items addressing work or school, social life, and family life, with two additional items on sleep difficulties and economic life. Participants were also asked about STIs they had contracted within the past 6 months or more than 6 months ago. Participants were also asked about their experiences with induced legal abortion, a sensitive topic, with an option to decline to answer.

#### Comorbid conditions

The Patient Health Questionnaire (PHQ-9)^8^ was used to assess depressive symptoms over the past two weeks. The Generalized Anxiety Disorder 7-item scale (GAD-7)^9^ was used to assess anxiety symptoms over the past two weeks. The Japanese version of the Adult Attention-deficit Hyperactivity Disorder Self-Report Scale (ASRS)^10^ was used to assess Adult Attention-deficit Hyperactivity Disorder (ADHD) symptoms. The scale includes 2 subscales: inattention and hyperactivity-impulsivity, each comprising nine items.

#### Treatment-seeking

Treatment-seeking behavior for various psychological and health problems was explored. Participant answered from a list of various psychological and health problems.

### Data analysis

Participants with a CSBD-19 score of 51 or higher were classified as high-risk, based on criteria from previous studies.^3^ Comparisons between high- and low-risk groups were performed using Welch’s *t*-test for continuous variables and the chi-squared test for categorical variables. Pearson’s correlation coefficients were calculated to examine associations between CSBD-19 scores and continuous variables. All statistical analyses were conducted using JASP Version 0.18.3 (JASP Team, Amsterdam, Netherlands).

## Results

### Associations with socio-demographic factors

Demographic details for the 1094 participants are presented in Tables 1. Twenty-eight participants (2.56%) were categorized as high risk, while 1066 (97.44%) were low risk based on the CSBD-19 threshold (Table 1). The chi-squared test indicated that heterosexual cisgender men were overrepresented in the high-risk group, whereas heterosexual cisgender women were predominant in the low-risk group (*P* = .010). No significant association was found for sexual and gender minorities from the residual analysis.

**Table 1:** Descriptive statistics and comparison of relevant variables based on CSBD-19 thresholds.

			**Total**		**Low-risk**		**High-risk**				
**Variables**			***N* = 1094**		**(*n* = 1066; 97.44%)**		**(*n* = 28; 2.56%)**		**Test statistic**	***P* value**	**Cramer’s *V*/Cohen’s*d***
	***Min***	***Max***	***n*/*Mean***	**%/*SD***	***n*/*Mean***	**%/*SD***	***n*/*Mean***	**%/*SD***			
Age (*Mean*, *SD*)	18	59	39.9	11.6	40	11.62	36.36	11.81	*t* (28.39) = 1.61	.118	*d* = 0.31
**Gender and sexual orientation**									χ^2^ (2) = 9.15	.010	*V* = .09
Heterosexual cisgender men			478	43.7%	460	43.2%	18	64.3%			
Heterosexual cisgender women			461	42.1%	457	42.9%	4	14.3%			
Gender and sexual minorities			155	14.2%	149	14.0%	6	21.4%			
**Income**									χ^2^ (3) = 0.26	.967	*V* = .02
Less than 4 million yen			299	27.3%	292	27.4%	7	25.0%			
More than 4 million and less than 6 million yen			200	18.3%	194	18.2%	6	21.4%			
More than 6 million yen			373	34.1%	364	34.1%	9	32.1%			
Do not know/not want to answer			222	20.3%	216	20.3%	6	21.4%			
**Employment status**									χ^2^ (3) = 5.40	.145	*V* = .07
Company employees, civil servants, executives, self-employed, and freelancers			523	47.8%	507	47.6%	16	57.1%			
Temporary and part time employees			253	23.1%	251	23.5%	2	7.1%			
Students			83	7.6%	79	7.4%	4	14.3%			
Homemaker, unemployed, and others			235	21.5%	229	21.5%	6	21.4%			
**Relationship status**									χ^2^ (4) = 0.29	.990	*V* = .02
Single			400	36.6%	390	36.6%	10	35.7%			
In a relationship			104	9.5%	101	9.5%	3	10.7%			
Married			504	46.1%	491	46.1%	13	46.4%			
Separated			57	5.2%	56	5.3%	1	3.6%			
Separated but now in a relationship			29	2.7%	28	2.6%	1	3.6%			
**Having children**									χ^2^ (1) = 0.01	.942	*V* = .002
Have children			437	40.0%	426	40.0%	11	39.3%			
Do not have child			657	60.1%	640	60.0%	17	60.7%			
**Education**									χ^2^ (2) = 0.64	.728	*V* = .02
High school			364	33.3%	356	33.4%	8	28.6%			
Some college			225	20.6%	220	20.6%	5	17.9%			
University or higher			505	46.2%	490	46.0%	15	53.6%			
**Residence**									χ^2^ (3) = 1.26	.740	*V* = .03
Capital			137	12.5%	132	12.4%	5	17.9%			
Government ordinance designated city			297	27.2%	289	27.1%	8	28.6%			
Core cities and special case cities			273	25.0%	268	25.1%	5	17.9%			
Cities, towns and village			387	35.4%	377	35.4%	10	35.7%			
**Living situation**									χ^2^ (2) = 0.46	.795	*V* = .02
Living alone			228	20.8%	222	20.8%	6	21.4%			
Living with someone but has private space			490	44.8%	476	44.7%	14	50.0%			
Living with someone but has not private space			376	34.4%	368	34.5%	8	28.6%			
Frequency of visiting sex establishments^a^	1	11	1.15	0.82	1.14	0.75	1.75	2.17	*t* (27.17) = 1.49	.148	*d* = 0.38
Frequency of telephone sex^a^	1	11	1.07	0.63	1.06	0.55	1.57	1.97	*t* (27.11) = 1.38	.180	*d* = 0.36
Frequency of cybersex^a^	1	11	1.13	0.92	1.12	0.87	1.61	2.03	*t* (27.26) = 1.27	.214	*d* = 0.31
Frequency of live streaming sex^a^	1	11	1.18	1.09	1.16	1.02	1.96	2.58	*t* (27.22) = 1.65	.111	*d* = 0.41
Time of pornography use in one session^b^	1	18	3.29	3.39	3.24	3.34	5.29	4.38	*t* (27.83) = 2.46	.020	*d* = 0.53
Time of masturbation in one session^b^	1	18	3.32	3.07	3.22	2.94	7.07	5	*t* (27.49) = 4.06	<.001	*d* = 0.94
Time of telephone sex in one session^b^	1	15	1.17	1.15	1.15	1.07	2	2.84	*t* (27.20) = 1.59	.125	*d* = 0.40
Time of cybersex in one session^b^	1	14	1.16	1.21	1.13	1.08	2.39	3.44	*t* (27.14) = 1.94	.063	*d* = 0.50
Longest duration of pornography use in a day^b^	1	18	3.77	4.41	3.7	4.37	6.25	5.22	*t* (28.01) = 2.56	.016	*d* = 0.53
Longest duration of masturbation in a day^b^	1	18	4.04	4.14	3.93	4.05	8.43	4.93	*t* (27.97) = 4.80	<.001	*d* = 1.00
Longest duration of telephone sex in a day^b^	1	18	1.14	1.14	1.12	1.04	1.96	3.1	*t* (27.16) = 1.45	.159	*d* = 0.37
Longest duration of cybersex in a day^b^	1	18	1.12	1.1	1.1	0.98	1.96	3.1	*t* (27.14) = 1.47	.152	*d* = 0.38
Overall quality of life	1	5	3.18	1.02	3.19	1.02	2.86	1.24	*t* (27.96) = 1.40	.173	*d* = 0.29
Satisfaction with health	1	5	3.16	1.01	3.17	1	2.68	1.19	*t* (28.02) = 2.19	.037	*d* = 0.45
Satisfaction with daily physical activities	1	5	3.43	0.96	3.44	0.96	3.07	1.09	*t* (28.12) = 1.78	.086	*d* = 0.36
Satisfaction with accessibility to health services	1	5	3.29	0.84	3.29	0.83	3.29	0.98	*t* (28.04) = 0.03	.978	*d* = 0.01
Satisfaction with oneself	1	5	2.94	1.05	2.94	1.04	2.71	1.21	*t* (28.06) = 0.98	.334	*d* = 0.20
Satisfaction with relationships in the workplace and school	1	5	3.17	0.91	3.18	0.9	3	1.12	*t* (27.93) = 0.83	.412	*d* = 0.18
Satisfaction with friendships	1	5	3.3	0.92	3.31	0.91	2.93	1.3	*t* (27.69) = 1.54	.136	*d* = 0.34
Satisfaction with family relationships	1	5	3.29	1.04	3.31	1.03	2.68	1.25	*t* (27.97) = 2.64	.013	*d* = 0.55
Satisfaction with partner (low-risk; *n* = 620, high-risk; *n* = 17)	6	24	17.53	4.64	17.6	4.61	15	4.87	*t* (16.80) = 2.17	.044	*d* = 0.55
Satisfaction with overall sex life	1	5	2.91	1	2.92	0.99	2.61	1.29	*t* (27.85) = 1.28	.212	*d* = 0.27
Satisfaction with sex life with partner (low-risk; *n* = 620, high-risk; *n* = 17)	1	5	3.05	1.04	3.06	1.03	2.88	1.27	*t* (16.59) = 0.56	.583	*d* = 0.15
Negative consequences work or school	0	10	0.18	0.87	0.15	0.79	1.29	2.21	*t* (27.18) = 2.71	.011	*d* = 0.68
Negative consequences social or leisure life	0	10	0.19	0.91	0.14	0.72	2.25	2.86	*t* (27.09) = 3.90	<.001	*d* = 1.01
Negative consequences family life	0	10	0.16	0.84	0.13	0.72	1.5	2.44	*t* (27.12) = 2.97	.006	*d* = 0.76
Negative consequences sleep life	0	10	0.31	1.11	0.26	0.95	2.32	3.12	*t* (27.13) = 3.50	.002	*d* = 0.9
Negative consequences economic life	0	10	0.25	1.05	0.21	0.96	1.61	2.51	*t* (27.21) = 2.93	.007	*d* = 0.73
Abortion histories (*n*, %)									χ^2^ (2) = 0.19	.912	*V* = .01
None			955	87.3%	931	87.3%	24	85.7%			
Experienced			113	10.3%	110	10.3%	3	10.7%			
Did not answer			26	2.4%	25	2.3%	1	3.6%			
**STIs in 6 months (*n*, %)**									χ^2^ (2) = 8.971	.011	*V* = .09
None			991	90.6%	970	91.0%	21	75.0%			
Experienced			21	1.9%	19	1.8%	2	7.1%			
Did not answer			82	7.5%	77	7.2%	5	17.9%			
**STIs in 6 more ago (*n*, %)**									χ^2^ (2) = 14.53	<.001	*V* = .12
None			955	87.3%	937	87.9%	18	64.3%			
Experienced			58	5.3%	53	5.0%	5	17.9%			
Did not answer			81	7.4%	76	7.1%	5	17.9%			
PHQ-9	0	27	6.23	6.34	6.1	6.21	11.39	8.75	*t* (27.72) = 3.18	.004	*d* = 0.70
GAD-7	0	21	4.42	5.35	4.3	5.24	9	7.46	*t* (27.70) = 3.31	.003	*d* = 0.73
ASRS total	0	72	16.86	12.05	16.42	11.52	33.79	18.36	*t* (27.56) = 4.98	<.001	*d* = 1.13
ASRS inattention	0	36	9.91	6.69	9.71	6.47	17.86	9.48	*t* (27.67) = 4.52	<.001	*d* = 1.00
ASRS hyperactivity/impulsivity	0	36	6.95	6.02	6.71	5.73	15.93	9.39	*t* (27.53) = 5.17	<.001	*d* = 1.18
**Potential of ADHD**									χ^2^ (1) = 48.74	< .001	*V* = .22
Not potential of ADHD			988	90.3%	974	91.37%	14	50%			
High potential of ADHD		14	106	9.7%	92	8.63%	14	50%			

Abbreviations: ASRS, Adult Attention-deficit Hyperactivity Disorder Self-Report Scale. CSBD-19, Compulsive Sexual Behavior Disorder Scale-19. GAD-7, Generalized Anxiety Disorder 7-item scale. PHQ-9, Patient Health Questionnaire-9. STIs, sexually transmitted infections.

a 1: never, 2: once a year, 3: 2-6 times a year, 4: 7-11 times a year, 5: once a month, 6: 2-3 times a month, 7: Once a week, 8: 2-3 times a week, 9: 4-5 times a week, 10: 6-7 times a week, 11: more than 8 times during a week.

b 1: None, 2: 5 minutes, 3: 10 minutes, 4: 15minutes, 5: 20minutes, 6: 25minutes, 7: 30minutes, 8: 35 minutes, 9: 40minutes, 10: 45minutes, 11: 50minutes, 12: 55minutes, 13: 1 hour, 14: 1 hour and a half, 15: 2 hour, 16: 3 hour, 17: 4 hour, 18: 5 hours or more.

^***^ *P* < .001, ^**^P < .01, ^*^P < .05.

### Characteristics of individuals at high risk of experiencing CSBD

The high-risk group spent significantly more time on pornography (*d* = 0.53) and masturbation (*d* = 0.94) per session than the low-risk group. Additionally, the high-risk group reported longer maximum duration of pornography use (*d* = 0.53) and masturbation (*d* = 1.00) within the last year. Regarding QOL, the high-risk group reported lower satisfaction with health (*d* = -0.45), family relationships (*d* = −0.55), and partner relationships (*d* = −0.55) compared to the low-risk group. The high-risk group experienced more adverse daily life consequences than the low-risk group, with large effect sizes for leisure (*d* = −1.01) and sleep (*d* = −0.90). They also reported higher rates of STIs in the last 6 months and the six months preceding. Higher levels of depressive (*d* = 0.70), anxiety (*d* = 0.73), and ADHD symptoms (*d* = 1.13) were seen in the high-risk group, with inattention (*d* = 1.00) and hyperactivity/impulsivity (*d* = 1.18) significantly elevated. The ASRS screener indicated a higher likelihood of ADHD in the high-risk group. In addition, three high-risk participants (10.71%) sought treatment for CSB (Supplementary Material).

Correlation coefficients were calculated between CSBD-19 score and continuous variables to explore bivariate associations (Table 2). Age was negatively correlated with the CSBD-19 score (*r* = −0.12) with a small effect size. Frequency, average time per session, and the longest duration of sexual activity were positively associated with the CSBD-19 score, though effect sizes were small. The CSBD-19 score was also negatively correlated with overall QOL (*r* = −0.10), daily living activities (*r* = −0.08), and self-satisfaction (*r* = -0.09), all showing trivial effects. Similar negative correlations were found for quality of friendships (*r* = -0.11), overall sex life satisfaction (*r* = −0.13), and satisfaction with a partner (*r* = −0.12).

**Table 2:** Correlation between CSBD-19 and each variable.

	**Total**		**Heterosexual** **cisgender men**		**Heterosexual** **cisgender women**		**Gender and** **Sexual minorities**	
Variables	*N* = 1094		*n* = 478		*n* = 461		*n* = 155	
	*r*	*P* value	*r*	*P* value	*r*	*P* value	*r*	*P* value
Age	−.12	<.001^***^	−.10	.034^*^	−.16	<.001^***^	−.15	.066
Frequency of visiting sex establishments^a^	.15	<.001^***^	.12	.006^**^	.08	.093	.19	.021^*^
Frequency of telephone sex^a^	.15	<.001^***^	.17	<.001^***^	.09	.060	.23	.005^**^
Frequency of cybersex^a^	.14	<.001^***^	.13	.006^**^	.10	.039^*^	.15	.065
Frequency of live streaming sex^a^	.10	<.001^***^	.13	.004^**^	.03	.595	−.02	.804
Time of pornography use in one session^b^	.28	<.001^***^	.20	<.001^***^	.20	<.001^***^	.21	.010^**^
Time of masturbation in one session^b^	.34	<.001^***^	.26	<.001^***^	.25	<.001^***^	.31	<.001^***^
Time of telephone sex in one session^b^	.18	<.001^***^	.12	.007^**^	.26	<.001^***^	.20	.013^*^
Time of cybersex in one session^b^	.17	<.001^***^	.15	<.001^***^	.20	<.001^***^	.17	.035^*^
Longest time of pornography use in a day^b^	.29	<.001^***^	.23	<.001^***^	.12	.008^**^	.28	<.001^***^
Longest time of masturbation in a day^b^	.36	<.001^***^	.31	<.001^***^	.16	<.001^***^	.36	<.001^***^
Longest time of telephone sex in a day^b^	.15	<.001^***^	.13	.005^**^	.20	<.001^***^	.17	.033^*^
Longest time of cybersex in a day^b^	.11	<.001^***^	.12	.010^*^	.08	.109	.13	.096
Overall quality of life	−.10	.002^**^	−.09	.045^*^	−.08	.073	−.01	.883
Satisfaction with health	−.08	.011^*^	−.13	.005^**^	−.04	.379	.04	.617
Satisfaction with daily physical activities	−.08	.006^**^	−.14	.003^**^	−.07	.150	.00	.985
Satisfaction with accessibility to health services	.00	.929	−.05	.245	.01	.838	.13	.118
Satisfaction with oneself	−.09	.004^**^	−.07	.157	−.06	.177	−.07	.383
Satisfaction with relationships in the workplace and school	−.05	.083	−.11	.020^*^	−.01	.918	.09	.268
Satisfaction with friendships	−.11	<.001^***^	−.12	.008^**^	−.09	.063	−.03	.674
Satisfaction with family relationships	−.12	<.001^***^	−.18	<.001^***^	−.06	.197	.03	.693
Satisfaction with partner (*n* = 637, hetero cis men = 250, hetero cis women = 325, gender and sexual minorities = 62)	−.10	.014^*^	−.16	.010^*^	< .01	.969	−.15	.239
Satisfaction with sex life with partner (*n* = 637, hetero cis men = 250, hetero cis women = 325, gender and sexual minorities = 62)	−.12	.002^**^	−.10	.134	−.02	.751	−.21	.096
Satisfaction with overall sex life	−.13	<.001^***^	−.11	.013^*^	−.07	.135	−.05	.570
Negative consequences work or school	.32	<.001^***^	.37	<.001^***^	.11	.014^*^	.32	<.001^***^
Negative consequences social or leisure life	.41	<.001^***^	.46	<.001^***^	.19	<.001^***^	.43	<.001^***^
Negative consequences family life	.30	<.001^***^	.34	<.001^***^	.16	<.001^***^	.37	<.001^***^
Negative consequences sleep life	.42	<.001^***^	.47	<.001^***^	.23	<.001^***^	.43	<.001^***^
Negative consequences economic life	.37	<.001^***^	.40	<.001^***^	.19	<.001^***^	.35	<.001^***^
PHQ-9	.20	<.001^***^	.23	<.001^***^	.22	<.001^***^	.19	.016^*^
GAD-7	.24	<.001^***^	.28	<.001^***^	.27	<.001^***^	.22	.006^**^
ASRS total	.31	<.001^***^	.39	<.001^***^	.29	<.001^***^	.22	.005^**^
ASRS inattention	.27	<.001^***^	.33	<.001^***^	.24	<.001^***^	.20	.012^*^
ASRS Hyperactivity/impulsivity	.33	<.001^***^	.40	<.001^***^	.30	<.001^***^	.23	.004^**^

Abbreviations: ASRS, Adult Attention-deficit Hyperactivity Disorder Self-Report Scale. CSBD-19, Compulsive Sexual Behavior Disorder Scale-19. GAD-7, Generalized Anxiety Disorder 7-item scale. PHQ-9, Patient Health Questionnaire-9. STIs, sexually transmitted infections.

a 1: never, 2: once a year, 3: 2-6 times a year, 4: 7-11 times a year, 5: once a month, 6: 2-3 times a month, 7: Once a week, 8: 2-3 times a week, 9: 4-5 times a week, 10: 6-7 times a week, 11: more than 8 times during a week.

b 1: None, 2: 5 minutes, 3: 10 minutes, 4: 15minutes, 5: 20minutes, 6: 25minutes, 7: 30minutes, 8: 35 minutes, 9: 40minutes, 10: 45minutes, 11: 50minutes, 12: 55minutes, 13: 1 hour, 14: 1 hour and a half, 15: 2 hour, 16: 3 hour, 17: 4 hour, 18: 5 hours or more.

^***^ *P* < .001, ^**^P < .01, ^*^P < .05.

## Discussion

This study explored the characteristics and treatment-seeking behaviors of individuals at high risk of experiencing CSBD in a national probability web-based sample of Japanese adults aged 18-59. Due to its cross-sectional nature, causal relationships cannot be determined. Cisgender heterosexual women were less likely to be at high risk of experiencing CSBD compared to cisgender heterosexual men based on chi-squared analysis. High-risk individuals reported lower life satisfaction across specific domains compared to low-risk individuals. They also reported spending more time on masturbation and pornography per session, and longer maximum durations of pornography use and masturbation in a single day within the past year. High-risk individuals experienced more adverse consequences across various life areas compared to low-risk individuals. In this sample, 2.56% (*n* = 28) of participants were at high risk of experiencing CSBD. Given the relatively small sample size (*N* = 1094) and the limited number of individuals at high risk (*n* = 28), these results should be viewed as preliminary, and further research, particularly within the context of Japanese culture, is needed.

Although detailed statistical analysis was not feasible in this study, gender identity and sexual orientation differences may have influenced comparisons between high- and low-risk groups. The small sample of diverse gender identities and sexual orientations in this analysis is a limitation that should be considered. Future studies should clarify these factors when assessing CSBD.

## Conclusion

This study is significant in identifying the characteristics of individuals at high risk of experiencing CSBD in Japan, contributing much-needed non-Western data on the impact of gender identity and sexual orientation on CSB.^1^ As the longest duration of pornography use and masturbation showed significant differences between high- and low-risk groups, suggesting that binge-like behaviors warrant further investigation. High-risk individuals reported greater psychosocial impairment, including lower health and intimate relationship satisfaction, sleep disturbances, and more negative life consequences. Although this study has methodological limitations, including small sample size and limited measurement methods, these findings underscore the need for inclusive mental health support and further research, particularly among women and gender minorities.

## Supplementary Material

Supplemental_qfaf091

## Data Availability

The raw data supporting the conclusion of this article will be made available by the authors upon request.

## References

[1] Grubbs JB, Hoagland KC, Lee BN, et al. Sexual addiction 25 years on: a systematic and methodological review of empirical literature and an agenda for future research. Clin Psychol Rev. 2020;82:101925. 10.1016/j.cpr.2020.101925 33038740

[2] World Health Organization . ICD-11 for mortality and morbidity statistics. Accessed May 28, 2024. https://icd.who.int/browse11/l-m/en

[3] Okabe Y, Ito D. Properties of the compulsive sexual behavior disorder Scale-19 among nationally representative sample in Japan. Int J Ment Health Addiction. 2024;22:3709–3732. 10.1007/s11469-023-01077-z

[4] Development of the World Health Organization WHOQOL-BREF quality of life assessment. The WHOQOL group. Psychol Med. 1998;28(3):551–558. 10.1017/s0033291798006667 9626712

[5] Norton R . Measuring marital quality: a critical look at the dependent variable. J Marriage Fam. 1983;45(1):141. 10.2307/351302

[6] Rosen RC, Riley A, Wagner G, Osterloh IH, Kirkpatrick J, Mishra A. The international index of erectile function (IIEF): a multidimensional scale for assessment of erectile dysfunction. Urology. 1997;49(6):822–830. 10.1016/s0090-4295(97)00238-0 9187685

[7] Sheehan DV . The Anxiety Disease. Charles Scribner and SonsScribner; New York, 1983.

[8] Muramatsu K, Miyaoka H, Kamijima K, et al. Performance of the Japanese version of the patient health Questionnaire-9 (J-PHQ-9) for depression in primary care. Gen Hosp Psychiatry. 2018;52:64–69. 10.1016/j.genhosppsych.2018.03.007 29698880

[9] Muramatsu K, Miyaoka H, Muramatsu Y, et al. Validation and utility of a Japanese version of the GAD-7. Jpn J Psychosom Med. 2010;50(6):592. 10.15064/jjpm.50.6_592_2

[10] Takeda T, Tsuji Y, Kurita H. Psychometric properties of the Japanese version of the adult attention-deficit hyperactivity disorder (ADHD) self-report scale (ASRS-J) and its short scale in accordance with DSM-5 diagnostic criteria. Res Dev Disabil. 2017;63:59–66. 10.1016/j.ridd.2017.02.011 28260624

