# Supplementary material for: Factors associated with compulsive sexual behavior in a national probability web-based aged 18-59 years sample from Japan: a cross-sectional survey
Source: Sex Med. 2025 Nov 4;13(5):qfaf091. doi: 10.1093/sexmed/qfaf091 (PMC12586315; doi:10.1093/sexmed/qfaf091)
Supplement: Supplemental_qfaf091 [file supplemental_qfaf091.docx]

**Supplementary**

**Appendix Introduction**

**Advances in the diagnosis and definition of compulsive sexual behavior disorder (CSBD)**

Previously, no formal diagnosis existed for out-of-control sexual behaviors. Although hypersexual disorder^1^ was proposed for inclusion in the Diagnostic and Statistical Manual of Mental Disorders (DSM-5), it was excluded from both DSM-5 and DSM-5-Text Revision (DSM-5-TR).^2,3^ Conversely, the International Classification of Diseases, 11th Revision (ICD-11) classifies compulsive sexual behavior disorder (CSBD) as an impulse control disorder.^4^ Core diagnostic features include: sexual activity becoming central to daily life at the expense of health, self-care, and responsibilities; repeated failed attempts to control behavior; and continued engagement despite negative consequences or reduced satisfaction. The disorder typically presents as a long-term pattern lasting six months or more and cannot be explained by another mental disorder, medical condition, or substance use. However, distress arising solely from moral judgments or disapproval of an individual’s sexual urges and behaviors does not meet the criteria for CSBD. ICD-11 notes that CSBD manifests across diverse various behaviors, including partnered sex, masturbation, pornography use, cybersex, telephone sex, and other forms of repetitive sexual behavior.^4^ There is a need to establish a foundation for effective prevention and treatment of CSBD, as recognized by ICD-11.^5,6,7^

**Term CSB**

Following previous literature,^8^ we refer to the clinical disorder evaluated according to ICD-11guidelines as CSBD. We use the term CSB to describe symptoms and self-reported difficulties controlling sexual behavior. Additionally, we use “at high risk of experiencing CSBD” for individuals scoring above the threshold on the CSBD-19, a self-report measure developed based on ICD-11 criteria.^9^ Thus, being at risk differs from receiving a clinical diagnosis, which requires clinical evaluation.

**Cultural background and Japanese context**

An international study across 42 countries found that 4.8% of participants were at high risk of experiencing CSBD, indicating that individuals in non-Western societies are also affected.^10^ Cultural differences in attitudes toward sexuality^11–14^ may also influence treatment-seeking behaviors for CSB. For example, a Polish study found that individuals at high risk of experiencing CSBD experienced more frequent, difficult-to-control sexual thoughts (withdrawal) and greater need for stimulation (tolerance symptoms).^15^ Similarly, Iranian married women at high risk of experiencing CSBD had lower economic status, religiosity, and sexual health, as well as higher substance use, anxiety, depression, and obsessive-compulsive symptoms.^16^

Despite the underdeveloped nature of sex education in Japan, commercial sexual acts such as pornography are culturally accepted. ^17,18^ Sexual intercourse plays a relatively minor societal and individual role in Japan, compared to Western countries.^19^ Additionally, the number of single and sexually inexperienced adults in Japan has increased.^20,21^ In this context, some report significant daily-life problems related to difficulty controlling sexual behaviors, such as pornography use, as shown in a preliminary study among university students.^22^

**Sociodemographic factors associated with CSB**

Identifying sociodemographic factors associated with CSBD is crucial for targeting populations in need of preventive and therapeutic interventions. This approach enables early support for specific groups (e.g., certain age or socioeconomic backgrounds) and help identify individuals at risk of severe CSBD. A global survey reported that 8.17% of men, 2.42% of women, and 6.46% of gender-diverse individuals were at high risk for CSBD.^10^ Similarly, a Polish study reported that 6.25% of men and 3.17% of women were at high risk.^15^ CSB is more frequently reported among men.^23,24^ However, other sociodemographic factors remains under-researched. For example, a study in the U.S. found that individuals with lower education, lower or higher income, and those from racial/ethnic and sexual and gender minorities were more likely to report CSB.^25^ Additionally, a study of German-speaking residents found that younger men with migratory backgrounds were more likely to experience CSB,^26^ whereas an Iranian study found associations with lower education, unemployment, and substance use.^16^ Conversely, no significant sociodemographic differences were observed among men meeting hypersexual disorder criteria.^1,27^ These mixed findings underscore the need to explore CSB risk factors in diverse contexts, including Japan.

**Variety of repetitive sexual behaviors and longest duration of sexual behavior**

There is also a need to understand the variety of repetitive sexual behaviors associated with CSBD.^28^ Research has focused primarily on pornography use and masturbation, while behaviors such as visiting sex establishments, telephone sex, cybersex, and live-streaming sex, remain underexplored.^29–33^ Masturbation may not necessarily involve pornography. While masturbation may be negatively associated with happiness, pornography may have minimal positive or neutral effects on relational happiness,^34^ supporting their distinct evaluation. Recent studies also suggest that assessing the longest duration of sexual behavior episodes may better characterize CSB.^35,36^ Binge behavior often involving prolonged, has been linked to CSB and is analogous to patterns seen in substance use or excessive media consumption.^37–41^ Although definitions vary, binge pornography use may involve hours-long or repeated episodes within a day.^36,42^ Among treatment-seekers, most report binge-like masturbation and pornography patterns, which predict CSB.^35,36^

In Japan, the frequency and duration of pornography use are well documented.^43^ Surveys found that 84% of men and 36% of women use pornography. Among those aged 20–29, 59% of men and 17% of women use pornography at least once weekly, while 15% of men and 2% of women report daily use.^19^ However, the association between CSB and behaviors such as visiting sex establishments, telephone sex, cybersex, live-streaming sex, and the longest duration spent on masturbation and viewing pornography, remains unclear in Japan.

**Life satisfaction, Negative consequences, and Psychological co-morbidities**

Individuals with high levels of CSB report negative consequences, including reduced quality of life (QOL), sexual satisfaction^44,45^ They also experience a range of adverse outcomes, such as interpersonal, educational, occupational, legal, and health problems like exposure to sexually transmitted infections (STIs).^26,32,33,46–49^ Understanding the impact on well-being is essential for service provision.

Treatment and prevention require addressing co-morbidities that may be involved in the development and maintenance of the disorder, as well as other conditions that share presenting symptoms or features, such as patterns of out-of-control sexual behavior.^50,51^ Anxiety, depression, and attention-deficit hyperactivity disorder (ADHD) symptoms are often associated with CSB.^27,31,52–57^ Anxiety and depression may lead to engagement in sexual behavior as mood regulation.^53,56–59^ Impulsivity and sensation-seeking, characteristic of adult ADHD, also contribute to out-of-control sexual behavior.^58^ Moreover, patterns of impaired control over sexual impulses, urges, or behaviors are observed in individuals with bipolar disorder, those on medication for Parkinson's disease, individuals with substance use problems, and those with dementia or brain injury.^8,60–63^ Among those with high CSB levels, 44.1% report alcohol abuse and 22.1% report substance abuse or dependence.^60^ However, these associations remain insufficiently understood in Japan.

**Treatment-seeking behavior**

To facilitate treatment access, mental health systems and specialized services for CSBD must be developed.^7,64^ In an international study, 6.4% of individuals at high risk sought treatment.^10^ Despite growing data from Western countries,^45,64,65^ treatment barriers remain high. In one study of 1,298 pornography use men, 14% expressed interest in seeking treatment for pornography use.^64^­ Similarly, in a survey of 674 Polish women, 31% reported seeking treatment for CSB,^65^ and a German study found that 21% of women and 13% of men with problematic pornography use sought therapy.^45^ However, the prevalence of treatment-seeking for CSB in Japan remains unknown.

**Hypothesis of this study**

Given limited data from non-Western settings, this study examined associations between CSB, sociodemographic factors, life satisfaction, negative consequences, psychological co-morbidities, and treatment-seeking in a national probability web-based sample of the Japanese population. In Japan, lower educational attainment and economic status contribute to CSB, whereas higher incomes and full-time employment may increase stress, prompting maladaptive coping through sexual behavior.^66^ A Japanese study on PPU found higher prevalence among younger, single men,^43^ suggesting that CSB may also be more prevalent among this group. We also hypothesized that living in large cities or alone may associated with CSB, due to easier access to sexual content and privacy. Lastly, we hypothesized that heterosexual individuals would report lower CSB levels than sexual and gender minorities, given stress-related vulnerabilities.^67,68^

**Appendix Methods**

***Participants and procedures***

Registered users of the survey company could earn points redeemable for cash or merchandise by completing various surveys (http://www.cm-group.co.jp/). Interested users accessed the study URL, where they were informed of the study purpose and provided online consent by checking a box before completing the questionnaire. Participants received points equivalent to 10–100 yen upon completion. The survey company used technical measures to prevent fraudulent access, including removing users who impersonate others, fail to update account information, provide dishonest responses, or do not respond to verification emails. Although fraud cannot be completely ruled out, these measures reduce its likelihood. A total of 361 users declined participation at the consent stage. However, we could not determine how many users accessed the study URL or began but did not complete the questionnaire. A total of 1,537 participants completed the survey. Of these, 443 (28.8%) were excluded based on the exclusion criteria described below. Respondents were excluded if they provided incorrect responses to the two Directed Questions Scale^69^ (e.g., trap questions such as, “Please select the rightmost option.”) (*n* = 431), or if they did not meet the company’s quality control criteria (*n* = 2). Although the exact nature of these criteria is proprietary to the survey company, they generally exclude users who provide inconsistent or dishonest responses. The final analysis, 1,094 participants (*Mean* age = 39.9 ± 11.6 years) were included. Data were collected based on the proportionality of sex assigned at birth, age group, and residential areas in accordance with the 2020 Japanese Population Census statistics.^70^ Sexual orientation was not included in census data^70^ and was not therefore used for stratification. Based on previous CSBD-19 studies,^9^ we expected 0.0–5.5% of women and 4.2–7.0% of men to be at high risk of experiencing CSBD but did not predetermine a target sample size; recruitment was limited by budget.

Of the final sample, 541 (49.45%) identified as women, 532 (48.63%) as men, and 21 (1.92%) as other. Regarding sexual orientation, 85.83% (*n*=939) identified as heterosexual people, 1.10% (n=12) as homosexual people, 3.66% (*n*=40) as bisexual people, and 2.74% (n=30) as asexual people. Additionally, 3.84% (*n*=42) chose “did not want to disclose or could not decide,” and 2.83% (n=31) selected “did not understand the question.”

For the analysis, participants (*N* = 1,094) were categorized based on gender identity and sexual orientation: those whose gender identity matched their sex assigned at birth and whose sexual orientation was heterosexual were grouped as heterosexual cisgender men or heterosexual cisgender women. Those with non-cisgender or non-heterosexual orientation were operationally grouped together as sexual and gender minorities. Thus, the distribution of participants across these categories was as follows: 478 (43.69%) were heterosexual cisgender men, 461 (42.14%) were heterosexual cisgender women, and 155 (14.17%) were operationally categorized as sexual and gender minorities (i.e., those categorized as sexual and gender minorities included participants identifying as "others" in terms of gender, or selecting “homosexual,” “bisexual,” “asexual,” “did not want to disclose or could not decide,” or “did not understand the question for sexual orientation”). The latter included anyone identifying as a gender other than their sex assigned at birth or selecting a non-heterosexual orientation. Although we expected that the relationship with CSB would differ depending on specific sexual orientations and gender identities, we created these three groups to simplify the statistical analysis due to the small sample sizes in the various sexual and gender minority groups.

***Measures***

**Sociodemographic data.**

In this study, the measurements of sex assigned at birth, gender identity, and sexual orientation were based on the method developed by Hiramori and Kamano^18^ for measuring these variables within a Japanese cultural context. Specifically, participants were first asked about their sex assigned at birth (“1. Please answer the sex assigned at birth,” and the answer options were “Male” or “Female”). Next, participants were asked about their perception of their current gender (“2. Do you consider that your current gender is the same as your sex assigned at birth,” and the answer options were “same as the sex assigned at birth,” “Different gender” or “Have a sense of discomfort”). If participants selected “Different gender” or “Have a sense of discomfort,” they were then asked to identify the gender that most closely matched their perception (“3. The gender that is closest to your current recognition,” and the answer options were “Man,” “Woman” or “Other”). Regarding gender identity classification in this study, participants who answered “Male” in sex assigned at birth (i.e., “1. Please answer the sex assigned at birth”) and then answered “Man” in their current perception (i.e., “3. The gender that is closest to your current recognition”), for their current gender, or “Female” for sex assigned at birth and “woman” for their current gender, were operationally categorized as “Cis gender people.” Those who answered “Female” or sex assigned at birth and “Man” or “Other” for their current gender, or “Male” for sex assigned at birth and “Female” or “Other” for their current gender, were operationally categorized as “Other” in terms of gender identity. Regarding sexual orientation, participants were asked to respond to the question: “Please indicate the number that you think is closest to you,” with the following options: “Heterosexual people,” “Gay men, lesbian women, homosexual people,” “Bisexual people,” “Asexual people,” “did not want to disclose or could not decide,” or “did not understand the question.”

Participants’ income was assessed with the question: “Please select the answer which your current annual household income.” Responses were provided on an 11-point scale: (“less than 2 million yen”, “2 to 4 million yen”, “4 to 6 million yen”, “6 to 8 million yen”, “8 to 10 million yen”, “10 to 12 million yen”, “10 to 15 million yen”, “10 to 20 million yen”, “20 million or more yen”, “do not know,” or “not want to answer”), and for the analysis, income was classified into five categories (“less than 4 million yen”, “more than 4 million and less than 6 million yen”, “more than 6 million yen”, and “do not know/not want to answer”). Regarding relationship status, participants were asked to select the answer that best described their current relationship status. The options were “single” (“I have never been married in my life and I am currently single”), “in a relationship” (“I have never been married in my life and I am currently in a relationship”), “married” (“I am currently married”), “separated” (“I have been separated from my spouse (e.g., divorce, bereavement) and I do not have a specific partner currently”), or “separated but now in a relationship” (“I have been separated from my spouse (e.g., divorce, bereavement) and I am currently in a relationship with a new partner”). Regarding having children (“Do you have children?”), and the answers were “I have children” or “I don’t have children.” Regarding the level of education, a single item, “Please select your highest level of education,” and the answers were “lower secondary schools,” “upper secondary schools,” “upper secondary specialized training school,” “professional training college,” “professional and vocational junior college, junior college, or college of technology,” “undergraduate program, educational institutions operated by government ministries and agencies, or professional and vocational university,” or “graduate school or professional graduate school.” For analysis, education level was classified into three categories (c.f., “high school” includes lower secondary schools, upper secondary schools, and upper secondary specialized training school, “some college” includes professional training college, professional and vocational junior college, junior college, and college of technology, and “university or higher” include undergraduate program, educational institutions operated by government ministries and agencies, professional and vocational university, graduate school, and professional graduate school. Regarding residence, participants reported the specific city, town, or village where they lived. For analysis, we categorized the population size of the residential area according to Japanese cabinet orders.^70^ The categories were as follows: “capital” (i.e., Tokyo), “government ordinance designated city” (i.e., a city with a population of 500,000 or more), “core cities and special case cities” (i.e., a city with a population of 200,000 or more), and “cities, towns, and village” (i.e., neither capital, government ordinance designated city, nor core cities and special case cities). Regarding the living situation, participants first answered whether they lived with someone by selecting from the following options: “living alone,” “living with partner,” “with parents,” “with parents-in-laws,” “with children,” “with siblings,” “with grandparents,” “with grandchildren,” “with other relatives,” and/or “with other people.” Next, if participants did not live alone, they were asked whether they had their own private space (i.e., “Do you have a private space that is exclusively yours?”). The answer options are: “I have my own private space (e.g., my room).” or “I don't have my own private space.” Based on responses to these questions, participants were classified into three categories for analysis: “living alone,” “living with someone but has private space,” and “living with someone but has not private space.” Regarding employment status, participants selected from the following options: “company employees, civil servants,” “executives, self-employed, freelancers”, “temporary employee”, “part time employee”, “students”, “homemaker”, “unemployed,” or “others For analysis, these categories were grouped into four based on labor market participation and employment stability (i.e., “company employees, civil servants, executives, self-employed, and freelancers”, “temporary and part time employee”, “students” and “homemaker, unemployed, and others”).

**Compulsive Sexual Behavior.**

The Compulsive Sexual Behavior Disorder Scale-19 (CSBD-19)^9^ was developed in alignment with the ICD-11 diagnostic guidelines for CSBD and is composed of five factors: control factor includes 3 items related to the failure to control CSB (e.g., “I could not control my sexual cravings and desires.”), salience includes 3 items related to CSB becoming the central focus of one's life (e.g., “I would rather have had sex than to have done anything else.”), relapse factor includes 3 items related to unsuccessful efforts to reduce CSB (e.g., “I was not successful in reducing the amount of sex I had.”), dissatisfaction factor includes 3 items related to experiencing less or no satisfaction from sexual behaviors (e.g., “I had sex even when I did not enjoy it anymore.”), and negative consequences factor includes 7 items related to CSB generating clinically significant distress or impairment (e.g., “I did not accomplish important tasks because of my sexual behavior”). This factor includes 1 item covering general adverse consequences, 1 item addressing general neglect, and 5 items assessing specific negative consequences across domains such as work and school, relationships, personal feelings, health and personal care, and other important areas of functioning. In an online survey across 42 countries,^10^ the CSBD-19 demonstrated structural validity, as well as measurement invariance across language, country, gender, and sexual orientation based on Goodness-of-Fit Indices. It also showed acceptable internal consistency (α = 0.68–0.90, ω = 0.68–0.90) and correlations with theoretically relevant sexual behaviors, such as the frequency of pornography use. Similarly, in a Japanese online survey, ^71^ the Japanese version of the CSBD-19 demonstrated structural validity, measurement invariance between men and women, high internal consistency (α = 0.84–0.96, ω = 0.85–0.96), and concurrent validity with relevant sexual behaviors. In the current sample, McDonald's ω was 0.96, and Cronbach's α was 0.96. The scale includes 19 items rated from 1 (“totally disagree”) to 4 (“totally agree”), covering five factors: control, salience, dissatisfaction, and negative consequences. Based on a previous study,^71^ a score of 51 or more indicates a higher risk of experiencing CSBD in Japan.

**Sexual behaviors.**

Participants reported the frequency of sexual behaviors, including visiting sex establishments (“How often did you visit sex establishments/massage parlor (e.g., *soap land, Fashion health, pink salon*) in the past year?” Note: *soap land*, *fashion health*, and *pink salon* are terms for specific types of Japanese sexual establishments), telephone sex (“How often did you have telephone sex in the past year?”), cybersex (“How often did you have online sex using video calls and chats, or exchanging sexually explicit photos or videos, or messages over the internet in the past year?”), and live streaming sex (“How often have you streamed or posted sexually explicit images of yourself online?”) over the past 1 year rated on an 11-point scale (1 = “never” to 11 = “more than eight times during a week”). These sexual behavior questions were developed by the authors, based on the ICD-11 guidelines and Kafka,^1,4^ to explore behaviors that have been less empirically investigated, such as visiting sex establishments, telephone sex, cybersex, and live streaming sex, compared to pornography use and in-person consensual sex.^5,28,29^ The 11-point response scale was adapted from previous research.^9,72^

Participants also reported the average time spent on sexual behaviors such as pornography use, masturbation, telephone sex, and cybersex during a single session over the past year, using an 18-point scale (1 = “none,” 2 = “five minutes,” 3 = “ten minutes,” 4 = “fifty minutes,” 5 = “twenty minutes,” 6 = “twenty- five minutes,” 7 = “thirty minutes,” 8 = “thirty-five minutes,” 9 = “forty minutes,” 10 = “forty-five minutes,” 11 = “fifty minutes,” 12 = “fifty-five minutes,” 13 = “one hour,” 14 = “one hour and thirty minutes,” 15 = “two hours,” 16 = “three hours”, 17 = “four hours”, 18 = “five hours or more”). To measure patterns of sexual behavior that may be associated with binge sexual behavior, participants also indicated the longest duration spent on sexual behaviors in a single day over the past year, including pornography use, masturbation, telephone sex, and cybersex (e.g., “What was the longest duration of the time that you spent using pornography in a day within the last year?”), using an 18-point scale (1 = “none” to 18 = “five hours or more”). Notably, we did not measure the average time spent in one session or the longest duration for visiting sex establishments or live-streaming sex due to the interactive nature of these activities between sexual partners and the ambiguity of their start and end points. These items, developed for exploratory purposes, do not cover all sexual behaviors or have formal psychometric properties. Before answering the pornography-related questions, participants were provided with a definition of pornography used in other studies:^73–75^ Pornography (1) creates or elicits sexual thoughts, feelings, or behaviors, and (2) contains explicit images or descriptions of sexual acts involving the genitals (e.g., vaginal or anal intercourse, oral sex, or masturbation).

**Life satisfaction.**

Five items from the WHO Quality of Life-BREF^76^ were used to assess quality of life over the past month. Specifically, participants were asked about their health, daily physical activity, accessibility to health services, and self-satisfaction to gain insights into their overall health. Additionally, three items were added to assess satisfaction with relationships at work or school (“How satisfied are you with the relationships you have at work, school, or in your local community?”), friendships (“How satisfied are you with your friendships?”), and family relationships (“How satisfied are you with your relationships with family members other than your partner?”). Responses to these eight questions, including the five WHO Quality of Life-BREF items and the three additional relationship satisfaction items, were rated on a five-point Likert scale ranging from 1 = "very dissatisfied" to 5= "very satisfied."

The Quality Marriage Index^77,78^ (QMI) consists of six items rated on a four-point scale (ranging from 1= “does not apply at all" to 4 = "applies very well”), with established reliability and validity.^78^ A higher score indicates greater satisfaction with the relationship. Participants were presented with the adapted QMI via an online questionnaire technology, depending on their relationship status. Only participants with partners (*n* = 637; participants who reported being in a relationship, married, or separated but currently in a relationship) were presented with the adapted QMI. Participants without partners were not presented with the adapted QMI. In the current sample, McDonald's ω was 0.96, and Cronbach's α was 0.96. Two items from the International Index of Erectile Function were used to assess sexual life quality.^79,80^ Specifically, participants were asked: "How satisfied have you been with your overall sex life in the past four weeks?", and "How satisfied have you been with your sexual relationship with your partner?” on a five-point scale ranging from 1 (very dissatisfied) to 5 (very satisfied). Participants without a partner were instructed to select (‘If you have not had a partner at least once in the last four weeks, please select “Neither agree nor disagree”’). In the analyses, the measure of sexual satisfaction with a partner and the adapted QMI were used for participants who had a partner (*n* = 637; participant in a relationship, a participant in married, and participants in separated but now in a relationship).

**Negative consequences.**

To assess negative consequences in daily life, five questions modified from the Sheehan Disability Scale,^81,82^ were used. These included three items addressing work or school, social life, and family life, and two additional items on sleep difficulties and economic life. To evaluate the negative impact of sexual activity, participants were asked: “How much did your sexual behavior negatively affect you?” Responses were rated on a scale from 0 = “no problems” to 10 = “extreme problems.” This question was asked for each of the five areas mentioned above (i.e., work or school, social life, family life, sleep, and economics). Participants were also asked about STIs they had contracted within the past 6 months or more than 6 months ago (This refers to when you found out you were infected, not when you were actually infected). Although many may not know exactly when they contracted an STI, the survey asked participants to select the appropriate option from a list. The survey asked participants to select the appropriate option from a list, including “never experienced,” “genital chlamydia infection,” “genital herpes,” “genital warts,” “syphilis,” “gonorrhea,” “hepatitis B,” “HIV/AIDS,” “others,” and “prefer not to answer.” For the analysis of STI, few people were affected by each type of sexually transmitted infection, and the participants were grouped into three: “individuals had a sexually transmitted infections”, “individuals did not”, and “individuals did not answer”. Participants were also asked about their experiences with induced legal abortion: “Please select the option that applies to you regarding your own experience of having an induced legal abortion or (if you are a man) the number of times your sexual partner had an induced legal abortion due to not your using contraception despite not wanting to get pregnant.” Responses ranged from 1 (never) to 6 (5 or more times), with “do not answer.” It is important to note that the wording of the abortion-related question may imply heteronormativity, which was an oversight. Future research should consider diverse sexual orientations and gender identities and adopt more inclusive language. For analysis, participants were categorized into three groups: those with no induced legal abortion experience, those who had had an induced legal abortion, and those who did not answer, regardless of the number of times they had an induced legal abortion.

**Comorbid conditions.**

The Patient Health Questionnaire (PHQ-9)^83,84^ consists of nine items (e.g., “Little interest or pleasure in doing things”) rated on a scale from 0 (not at all) to 3 (nearly every day). Scores range from 0 to 27, with higher scores indicating more severe depressive symptoms. The PHQ-9 was translated from English to Japanese by Muramatsu et al,^84^ and the back-translation was confirmed for accuracy by Spitzer, the original developer of the PHQ-9. The Japanese version of the PHQ-9 has been validated for assessing depression in patients diagnosed with major depressive disorder through psychiatric interviews.^84^ Internal consistency has been demonstrated to be adequate in Japanese general population samples (Cronbach's α was .89).^85^ In the current sample, McDonald's ω was .92, and Cronbach's α was .92.

The Generalized Anxiety Disorder 7-item scale (GAD-7) ^86,87^ consists of seven items (e.g., “Feeling nervous, anxious or on edge”), which is rated on a four-point scale from 0 (not at all) to 3 (nearly every day). Scores range from 0–21, with higher scores indicating more severe anxiety symptoms. The GAD-7 was translated from English to Japanese by Muramatsu et al, ^88^ using the back-translation method, with guidance from Spitzer and Kroenke, the developers of the GAD-7, to confirm the accuracy of the translation. The Japanese version of the GAD-7 has been validated for use in assessing generalized anxiety disorder in patients diagnosed via psychiatric interviews.^88^ A survey of Japanese adults further confirmed that one- and two-factor structures can be applied and that the scale demonstrates measurement invariance across three groups: those with self-reported anxiety disorder, those with self-reported anxiety disorder and major depressive disorder, and those without self-reported psychiatric diagnoses. Convergent and discriminant validity have also been demonstrated.^89^ Internal consistency has been found to be adequate in general Japanese samples (Cronbach's α = 0.91).^25^ In the current sample, McDonald's ω was 0.94, and Cronbach's α was 0.94.

The Adult Attention-Deficit Hyperactivity Disorder Self-Report Scale (ASRS) ^90^ consists of 18 items (e.g., “How often do you have trouble wrapping up the final details of a project once the challenging parts have been done?”) rated on a five-point scale (ranging from 0 = "never" to 4 = "very often"). This study used the Japanese version of the ASRS, which was translated by Takeda et al^91^ in accordance with the Principles of Good Practice guidelines for translating scales proposed by the International Society for Pharmacoeconomics and Outcomes Research (ISPOR) task force for Translation and Cultural Adaptation (TCA).^92^ The ASRS also includes a 6-item shortened version, known as the ASRS Screener, which can be used to screen for ADHD based on four or more specific item responses.^91^ In previous studies involving adults with ADHD, non-ADHD adults, non-clinical adults, and university students, the Japanese version of the ASRS demonstrated good internal consistency, with Cronbach's α values around 0.80. Test-retest reliability over a two-week period was also confirmed. Additionally, discriminant validity was established, with the ADHD group scoring higher than the other groups. Concurrent and divergent validity were verified through correlations with other ADHD scales and with depressive symptoms.^91^ For the current sample, McDonald's ω was 0.95 and Cronbach's α was 0.95 for the full ASRS. For the inattention subscale, ω = 0.93 and α = 0.93, and for hyperactivity-impulsivity, ω = 0.91 and α = 0.91.

**Treatment-Seeking.**

Treatment-seeking behavior for various psychological and health problems was explored through the following question: “Please let us know if you are currently seeking any kind of treatment, support, or rehabilitation at a hospital, counseling institution, or self-help group.” Participant responses were as follows: “I have not sought anything”, “I don't know what I have psychological and health problems, and/or I don't want to answer.” “dementia,” “brain injury,” “Parkinson’s disease,” “bipolar disorder,” “compulsive sexual behavior,” “alcohol addiction,” “drug addiction,” “gambling disorder,” “eating disorder,” and “post-traumatic stress disorder.” Although this questionnaire included formal sources of treatment (e.g., hospitals) and informal sources (e.g., self-help groups), the priority in this study was to capture a broad range of mental health service access. Given that no previous studies have investigated treatment-seeking behavior for CSB in Japan, our aim was to measure access to various forms of treatment, regardless of the specific type of service.

***Data analysis***

Data were summarized as *Means* ± *SD* for continuous variables and *N* (%) for categorical variables. Cohen's *d* values were interpreted as follows: <0.20, trivial; 0.20–0.50, small; 0.50–0.80, medium; and ≥0.80, large.^93^ Correlation coefficients (*r*) were interpreted as: <0.10, trivial; 0.10–0.30, small; 0.30–0.50, medium; and ≥0.50, large.^94^ Statistical significance was established at a two-sided *P*-value of 0.05. For analyses involving relationship and sexual satisfaction with partners, only participants who had partners (*n* = 637) were included. All other analyses included partnered and unpartnered participants (*N* = 1,094). Pearson’s correlation coefficients were calculated to examine associations between CSBD-19 scores and continuous variables, stratified by gender identity and sexual orientation (i.e., heterosexual cisgender men, heterosexual cisgender women, and sexual and gender minorities). Correlation analyses explored relationships between CSBD-19 scores and other factors. No missing data were reported for any of the 1,094 participants analyzed, and all statistical analyses were conducted using JASP Version 0.18.3 (JASP Team, Amsterdam, Netherlands).^95^

**Appendix Results**

***Descriptive statistics of sexual behavior***

Frequency of visiting sex establishments, engaging in telephone sex, cybersex, and live streaming sex within the past year ranged from “never” to “once or twice a year.” The reported time spent on pornography use and masturbation per session ranged from 10–15 minutes, whereas cybersex and telephone sex sessions typically lasted 5–10 minutes. The longest duration spent on watching pornography and masturbating were 10–15 minutes and 15–20 minutes, respectively. Cybersex and phone sex had the longest durations of approximately 5–10 minutes (**Table 1 of Manuscript**).

***Characteristics of individuals at high risk of experiencing CSBD***

No significant differences were found in the frequency of visiting sex establishments, engaging in telephone sex, cybersex, or live-streaming sex based on Welch's *t*-test (**Table 1 of Manuscript**). Similarly, no significant differences were observed in the average time spent per session on telephone sex or cybersex. The low-risk group averaged 10–15 minutes per session for both activities, whereas the high-risk group spent 20–25 minutes on pornography and 30–35 minutes on masturbation. No significant differences were found in abortion histories between the groups.

Among high-risk participants, one sought treatment for brain injury and two for Parkinson's disease (**Table S1**). Three high-risk participants (10.71%) sought treatment for CSB, all of whom were heterosexual cisgender men.

| **Table S1** | | | | | | | |
| --- | --- | --- | --- | --- | --- | --- | --- |
| Treatment-seeking behaviors for compulsive sexual behavior and other health problems. | | | | | | | |
|  | Low-risk | |  | High-risk | |  |  |
| Variables | (*n* = 1066; 97.4%) | |  | (*n* = 28; 2.56%) | | Fisher's exact tests |  |
|  | *n* | % |  | *n* | % | *p* | Cramer's *V* |
| I have not sought anything | 968 | 91% |  | 19 | 67.9% | < .001 | .122 |
| I do not know what I have psychological and health problems and/or I do not want to answer. | 54 | 5.1% |  | 1 | 3.6% | 1.00 | .011 |
| Dementia | 1 | 0.1% |  | 0 | 0% | 1.00 | .005 |
| Brain injury | 0 | 0% |  | 1 | 3.6% | .026 | .187 |
| Parkinson’s disease | 1 | 0.1% |  | 2 | 7.1% | .002 | .213 |
| Bipolar disorder | 7 | 0.7% |  | 1 | 3.6% | .188 | .054 |
| Compulsive sexual behavior | 0 | 0% |  | 3 | 10.7% | < .001 | .324 |
| Alcohol addiction | 6 | 0.6% |  | 1 | 3.6% | .166 | .060 |
| Drug addiction | 4 | 0.4% |  | 0 | 0% | 1.00 | .010 |
| Gambling disorder | 2 | 0.2% |  | 1 | 3.6% | .075 | .102 |
| Eating disorder | 7 | 0.7% |  | 0 | 0% | 1.00 | .013 |
| Post-traumatic stress disorder | 21 | 2.0% |  | 2 | 7.1% | .115 | .057 |
| *Note*. The items were presented in a multiple-response format, and participants could select more than one option. As a result, the total percentage may exceed 100%. | | | | | | | |

**Appendix Discussion**

Based on the CSBD-19 cutoff for Japan, 2.56% of participants were classified as high risk of experiencing CSBD. This prevalence is lower than the 4.2% reported in a global survey of 42 countries but falls within the 1.6–16.7% range observed across different populations.^10^ It is important to note that the CSBD-19 is a screening tool, and formal diagnosis requires comprehensive evaluation, including clinical interviews by experts.

Our finding that women have a lower risk of CSBD, as shown by the chi-squared test, aligns with previous studies. ^23,24,65,67,68^ A weak correlation between age and CSB was also observed, supporting earlier findings of higher risks among younger individuals.^26^ However, no significant associations were found between high risk for CSBD and factors such as income, employment, relationship status, children, education, region, or residence type. Consistent with prior research, no significant association was found between relationship status and CSBD.^8^ However, past studies have linked single status to PPU, a specific form of CSB.^75,96^ Additionally, previous research has shown that abuse experiences can moderate the relationship between relationship status and CSB.^97,98^ Divorce or separation has been associated with more severe CSB symptoms in women compared to those married or in common-law relationships.^65^ These findings highlight the complexity of the relationship between relationship status and CSB.

No differences were found in the frequency of visiting sex establishments, telephone sex, cybersex, or live-streaming sex. However, individuals at high risk of experiencing CSBD reported longer maximum durations of pornography use and masturbation in a day compared to low-risk individuals, aligning with previous research.^35,36^ High-risk individuals also spent more time per session on masturbation and pornography, consistent with earlier findings.^26,30,33^ Although the high-risk group spent an average of 20–25 minutes per pornography session and 30–35 minutes per masturbation session, these amounts of time alone do not indicate CSBD. CSBD should not be assessed solely by the amount of sexual activity, as low-risk individuals may spend similar amounts of time on sexual behavior. The correlations reveal interesting patterns. Among heterosexual cisgender men and sexual and gender minorities, the strongest association with CSB was observed for the longest duration of pornography use and masturbation rather than the average time per session. This supports the idea that the longest pornography viewing sessions, potentially related to binge behavior in men, may better predict behavioral dysregulation.^35,36^ Conversely, for heterosexual cisgender women, average time per session on pornography and masturbation showed a stronger association with CSB. Previous studies on binge pornography and masturbation have primarily focused on men,^35,36^ and these results indicate that the relationship between binge sexual behaviors and CSB may differ in women.

Associations between CSB and various sexual behaviors (e.g., visiting sex establishments, telephone sex, cybersex, live-streaming sex) also varied by gender identity and sexual orientation. Heterosexual cisgender women showed weak or no associations between CSB and these sexual behaviors. It is possible that environmental factors, such as the lower accessibility of commercial sexual activities to women, may have influenced these associations. In Japan, for example, 48% of men and only 4% of women have ever used commercial sex worker services.^19^ The sex industry targeting women is less developed and widespread than that targeting men.^99^ These findings suggest that the relationship between time spent on sexual behaviors and the types of sexual behaviors engaged in may depend on gender identity and sexual orientation, highlighting the need for further research.

Individuals at high risk of experiencing CSBD reported lower satisfaction with their health and intimate relationships, including those with family and romantic partners. In heterosexual cisgender men, correlation analysis revealed a link between CSB and lower relationship satisfaction. Although this study was unable to determine a causal relationship, the causal relationship between CSB and relationship satisfaction is complex.^100^ Individuals may use pornography excessively or engage in multiple sexual partnerships to fulfill unmet interpersonal needs.^101–104^ Conversely, repetitive sexual behaviors can strain relationships with partners and family members.^46,47^

CSB was also linked to several negative life consequences, consistent with prior research.^46,47,105^ In this study, individuals at high risk of experiencing CSBD reported more experiences with STIs. However, since individuals may not know the source of their STI or recall details of their diagnosis, the data on STI experiences may be unreliable. Therefore, these results should be interpreted as preliminary. Future studies should examine sexual risk behaviors and safer sex practices within the context of CSB. Correlation analyses identified sleep disturbances and negative consequences in social and leisure activities as strongly linked to CSB. Studies on PPU similarly suggest that pornography consumption disrupts sleep and leisure.^106^ These findings suggest that individuals with high CSB levels may benefit from mental health services addressing their negative experiences. ^107^

Depression, anxiety, and ADHD symptoms are also associated with CSB. Therefore, treatment may need to address co-morbid depression and anxiety. Correlational analysis showed that heterosexual cisgender men had stronger associations between CSB and ADHD symptoms compared to women and gender and sexual minorities, suggesting that ADHD’s role in CSB may vary by gender identity and sexual orientation. Previous studies have found ADHD symptoms linked to PPU in women.^52^ However, ADHD symptoms may not directly predict CSB. Instead, the impulsivity observed in CSB may be due to factors like risk-taking and lack of planning rather than ADHD itself.^108^ Nonetheless, the co-morbidity of ADHD and CSBD warrants further investigation, considering gender identity and sexual orientation and sexual behavior differences.^58^

Although 10.71% (*n* = 3) of individuals at high risk of experiencing CSBD sought treatment, the majority (89.29%; n = 25) did not. However, because the study is based on a small sample, these results are preliminary. Although large-scale data from other countries confirm that individuals seek treatment for CSB,^10^ expanding research is crucial to improving access to appropriate mental healthcare for those seeking CSB treatment.^7^ This study did not include a clinical group, making it unclear to what extent individuals with CSBD in Japan need treatment. Therefore, assessing the need for expanded mental health services for CSB in Japan is crucial. Further research should identify barriers to treatment-seeking behavior, such as stigma, limited public awareness that CSB is covered by mental health services, and insufficient knowledge among clinicians and counselors.^45,109^ Additionally, individuals at high risk of experiencing CSBD also included those seeking treatment for brain injury and Parkinson's disease. However, this finding should be interpreted cautiously due to the small sample size. It underscores the limitations of self-reported measures and emphasizes the need for comprehensive assessments, including diagnostic interviews, in clinical practice for CSBD.^8,110^

**Limitations**

This study has several limitations. First, the sample number of individuals at high risk and the underrepresentation of gender and sexual minorities limited subgroup analyses. Although efforts were made to include individuals of diverse gender identities and sexual orientations, the small sample size may have contributed to the underrepresentation of gender and sexual minorities. Future research should adopt methods that better capture the diversity within gender identity and sexual orientation. Furthermore, as 64.3% of high-risk individuals were heterosexual cisgender men, gender identity and sexual orientation may have influenced the differences between high-risk and non-high-risk groups. Future studies should increase statistical power by including larger samples of women and gender and sexual minorities to enhance generalizability. This study also used unbalanced groups of 1066 and 28 participants. Therefore, this study should be interpreted as a primary result for Japanese people.

Although participants' sex assigned at birth, age, and residential area matched the Japanese census, recruitment through an internet survey company and interest in answering sexual surveys may have influenced the findings’ generalizability. Participants were those who completed the web-based questionnaires as requested, and those who voluntarily withdrew were excluded. For example, individuals uncomfortable with sexual questions may have dropped out midway. Additionally, the sexual satisfaction questionnaire posed challenges for participants without partners, potentially biasing the results. Thus, the sample may represent individuals more comfortable discussing their sexuality. Future studies should explore which questions led to drop out to better understand trends in the sample.

Regarding the measures used, this study assessed pornography use, masturbation, visiting sex establishments, telephone sex, cybersex, and live-streaming sex, all common behaviors in individuals with CSB. However, future studies should also examine other behaviors, such as chemsex and group sex, which may also be relevant. Additionally, we used single-item or limited-item measures for QOL and negative consequences, which restricted the depth of our findings. Future research in Japan should use validated scales, such as the Hypersexual Behavior Consequence Scale, to capture the negative consequences of CSB more thoroughly.^47^ This study did not assess contraception-related behaviors, which are important for evaluating sexual risk. Future studies should explore the relationship between CSB and sexual risk behaviors, including contraceptive use. Although we examined psychological co-morbidities, we did not measure some important conditions, such as obsessive-compulsive, personality, and paraphilic disorders, which are relevant in CSBD, as mentioned in the ICD-11.^4^ Future research should further investigate these boundaries. Although this study examined access to mental health services for CSB, it did not differentiate between the types of treatment sources used. Since treatment approaches vary across providers like hospitals, community mental health centers, and self-help groups, future research should identify specific sources of support utilized by individuals with CSB. Lastly, the cross-sectional design of this study limited our ability to determine causality. Future longitudinal studies are needed to identify factors contributing to the onset and progression of CSBD.

**References in the supplementary**

1. Kafka MP. Hypersexual disorder: a proposed diagnosis for DSM-V. *Arch Sex Behav*. 2010;39(2):377-400. https://doi.org/10.1007/s10508-009-9574-7
2. American Psychiatric Association. *Diagnostic and statistical manual of mental disorders*. 5th ed. American Psychiatric Association Publishing; 2013. https://doi.org/10.1176/appi.books.9780890425596
3. American Psychiatric Association. *Diagnostic and statistical manual of mental disorders*. 5th ed., text rev. American Psychiatric Association Publishing; 2022. https://doi.org/10.1176/appi.books.9780890425787
4. World Health Organization. ICD-11 for mortality and morbidity statistics. Accessed May 28, 2024. https://icd.who.int/browse11/l-m/en
5. Grubbs JB, Hoagland KC, Lee BN, et al. Sexual addiction 25 years on: A systematic and methodological review of empirical literature and an agenda for future research. *Clin Psychol Rev*. 2020;82:101925. https://doi.org/10.1016/j.cpr.2020.101925
6. Reed GM, First MB, Billieux J, et al. Emerging experience with selected new categories in the ICD-11: complex PTSD, prolonged grief disorder, gaming disorder, and compulsive sexual behaviour disorder. *World Psychiatry*. 2022;21(2):189-213. https://doi.org/10.1002/wps.20960
7. Gola M, Potenza MN. Promoting educational, classification, treatment, and policy initiatives. *J Behav Addict*. 2018;7(2):208-210. https://doi.org/10.1556/2006.7.2018.51
8. Briken P, Bőthe B, Carvalho J, et al. Assessment and treatment of compulsive sexual behavior disorder: a sexual medicine perspective. *Sex Med Rev*. 2024;12(3):355-370. https://doi.org/10.1093/sxmrev/qeae014
9. Bőthe B, Potenza MN, Griffiths MD, et al. The development of the Compulsive Sexual Behavior Disorder Scale (CSBD-19): An ICD-11 based screening measure across three languages. J Behav Addict. 2020;9(2):247–258. https://doi.org/10.1556/2006.2020.00034
10. Bőthe B, Koós M, Nagy L, et al. Compulsive sexual behavior disorder in 42 countries: Insights from the International Sex Survey and introduction of standardized assessment tools. *J Behav Addict*. 2023;12(2):393–407. https://doi.org/10.1556/2006.2023.00028
11. Mestre-Bach G, Blycker GR, Actis CC, Brand M, Potenza MN. Religion, morality, ethics, and problematic pornography use. *Curr Addict Rep*. 2021;8(4):568–577. https://doi.org/10.1007/s40429-021-00388-2
12. Loughnan S, Fernandez S, et al. Exploring the Role of Culture in Sexual Objectification: A Seven Nations Study. *Int Rev Soc Psychol.* 2015;28: 125-152. http://www.cairn.info/revue-internationale-de-psychologie-sociale-2015-1-page-125.htm
13. Stephens T, Kamimura A, Yamawaki N, et al. Rape myth acceptance among college students in the United States, Japan, and India. *SAGE Open*. 2016;6(4):215824401667501. https://doi.org/10.1177/2158244016675015
14. Ho CC, Singam P, Hong GE, Zainuddin ZM. Male sexual dysfunction in Asia. *Asian J Androl*. 2011;13(4):537–542. https://doi.org/10.1038/aja.2010.135
15. Lewczuk K, Wizła M, Glica A, Potenza MN, Lew-Starowicz M, Kraus SW. Withdrawal and tolerance as related to compulsive sexual behavior disorder and problematic pornography use - Preregistered study based on a nationally representative sample in Poland. *J Behav Addict*. 2022;11(4):979–993. https://doi.org/10.1556/2006.2022.00076
16. Khayer E, Zarei R, Damghanian M, Bőthe B, Farnam F. Compulsive sexual behaviour in Iranian married women: Prevalence, sociodemographic, sexual, and psychological predictors across-country. *J Behav Addict*. 2024;13(2):495-505. https://doi.org/10.1556/2006.2024.00009
17. Fu H. The bumpy road to socialise nature: sex education in Japan. *Cult Health Sex*. 2011;13(8):903-915. https://doi.org/10.1080/13691058.2011.587894
18. Hiramori D, Kamano S. Asking about Sexual Orientation and Gender Identity in Social Surveys in Japan: Findings from the Osaka City Residents’ Survey and Related Preparatory Studies. SocArXiv. 2020;1-24. https://doi.org/10.31235/osf.io/w9mjn
19. Ghaznavi C, Ueda P, Okuhama A, Sakamoto H. Sexual Behaviors among Individuals Aged 20-49 in Japan: Initial Findings from a Quasi-Representative National Survey, 2022. *J Sex Res*. 2024;61(1):9-20. https://doi.org/10.1080/00224499.2023.2178614
20. Ghaznavi C, Sakamoto H, Nomura S, et al. The herbivore's dilemma: Trends in and factors associated with heterosexual relationship status and interest in romantic relationships among young adults in Japan-Analysis of national surveys, 1987-2015. *PLoS One*. 2020;15(11):e0241571. https://doi.org/10.1371/journal.pone.0241571
21. Ghaznavi C, Sakamoto H, Yoneoka D, Nomura S, Shibuya K, Ueda P. Trends in heterosexual inexperience among young adults in Japan: analysis of national surveys, 1987-2015. *BMC Public Health*. 2019;19(1):355. https://doi.org/10.1186/s12889-019-6677-5
22. Okabe Y, Takahashi F, Ito D. Problematic Pornography Use in Japan: A Preliminary Study Among University Students. *Front Psychol*. 2021;12:638354. https://doi.org/10.3389/fpsyg.2021.638354
23. Kowalewska E, Gola M, Kraus SW, Lew-Starowicz M. Spotlight on Compulsive Sexual Behavior Disorder: A Systematic Review of Research on Women. *Neuropsychiatr Dis Treat*. 2020;16:2025-2043. https://doi.org/10.2147/NDT.S221540
24. Kürbitz LI, Briken P. Is Compulsive Sexual Behavior Different in Women Compared to Men? J*ournal of Clinical Medicine*. 2021; 10(15):3205. https://doi.org/10.3390/jcm10153205
25. Dickenson JA, Gleason N, Coleman E, Miner MH. Prevalence of Distress Associated With Difficulty Controlling Sexual Urges, Feelings, and Behaviors in the United States. *JAMA Netw Open*. 2018;1(7):e184468. https://doi.org/10.1001/jamanetworkopen.2018.4468
26. Briken P, Wiessner C, Štulhofer A, et al. Who feels affected by "out of control" sexual behavior? Prevalence and correlates of indicators for ICD-11 Compulsive Sexual Behavior Disorder in the German Health and Sexuality Survey (GeSiD). *J Behav Addict*. 2022;11(3):900911. https://doi.org/10.1556/2006.2022.00060
27. Engel J, Veit M, Sinke C, et al. Same Same but Different: A Clinical Characterization of Men with Hypersexual Disorder in the Sex@Brain Study. *J Clin Med*. 2019;8(2):157. https://doi.org/10.3390/jcm8020157
28. Antons S, Brand M. Diagnostic and classification considerations related to compulsive sexual behavior disorder and problematic pornography use. *Curr Addict Rep*. 2021;8(3):452-457. https://doi.org/10.1007/s40429-021-00383-7
29. de Alarcón R, de la Iglesia JI, Casado NM, Montejo AL. Online Porn Addiction: What We Know and What We Don't-A Systematic Review. *J Clin Med*. 2019;8(1):91. https://doi.org/10.3390/jcm8010091
30. Reid RC, Carpenter BN, Hook JN, et al. Report of findings in a DSM-5 field trial for hypersexual disorder. *J Sex Med*. 2012;9(11):2868-2877. https://doi.org/10.1111/j.1743-6109.2012.02936.x
31. Castro-Calvo J, Gil-Llario MD, Giménez-García C, Gil-Juliá B, Ballester-Arnal R. Occurrence and clinical characteristics of Compulsive Sexual Behavior Disorder (CSBD): A cluster analysis in two independent community samples. *J Behav Addict*. 2020;9(2):446-468. https://doi.org/10.1556/2006.2020.00025
32. González-Bueso V, Santamaría JJ, Caro-Pérez O, et al. Compulsive Sexual Behavior Online and Non-online in Adult Male Patients and Healthy Controls: Comparison in Sociodemographic, Clinical, and Personality Variables. *Front Psychiatry*. 2022;13:839788. https://doi.org/10.3389/fpsyt.2022.839788
33. Wéry A, Vogelaere K, Challet-Bouju G, et al. Characteristics of self-identified sexual addicts in a behavioral addiction outpatient clinic. *J Behav Addict*. 2016;5(4):623-630. https://doi.org/10.1556/2006.5.2016.071
34. Perry SL. Is the Link Between Pornography Use and Relational Happiness Really More About Masturbation? Results From Two National Surveys. *J Sex Res*. 2020;57(1):64-76. https://doi.org/10.1080/00224499.2018.1556772
35. Lewczuk K, Lesniak J, Lew-Starowicz M, Gola M. Variability of Pornographic Content Consumed and Longest Session of Pornography Use Associated With Treatment Seeking and Problematic Sexual Behavior Symptoms. *Sex Med*. 2021;9(1):100276. https://doi.org/10.1016/j.esxm.2020.10.004
36. Wordecha M, Wilk M, Kowalewska E, Skorko M, Łapiński A, Gola M. "Pornographic binges" as a key characteristic of males seeking treatment for compulsive sexual behaviors: Qualitative and quantitative 10-week-long diary assessment. *J Behav Addict*. 2018;7(2):433-444. https://doi.org/10.1556/2006.7.2018.33
37. Escrivá-Martínez T, Herrero R, Molinari G, Rodríguez-Arias M, Verdejo-García A, Baños RM. Binge Eating and Binge Drinking: A Two-Way Road? An Integrative Review. *Curr Pharm Des*. 2020;26(20):2402-2415. https://doi.org/10.2174/1381612826666200316153317
38. Rolland B, Naassila M. Binge Drinking: Current Diagnostic and Therapeutic Issues. *CNS Drugs*. 2017;31(3):181-186. https://doi.org/10.1007/s40263-017-0413-4
39. Marmet S, Wicki M, Dupuis M, et al. Associations of binge gaming (5 or more consecutive hours played) with gaming disorder and mental health in young men. *J Behav Addict*. 2023;12(1):295-301. https://doi.org/10.1556/2006.2022.00086
40. Flayelle M, Maurage P, Di Lorenzo KR, Vögele C, Gainsbury SM, Billieux J. Binge-Watching: What Do we Know So Far? A First Systematic Review of the Evidence. *Curr Addict Rep*. 2020;7(1):44-60. https://doi.org/10.1007/s40429-020-00299-8
41. Cowlishaw S, Nespoli E, Jebadurai JK, Smith N, Bowden-Jones H. Episodic and Binge Gambling: An Exploration and Preliminary Quantitative Study. *J Gambl Stud*. 2018;34(1):85-99. https://doi.org/10.1007/s10899-017-9697-z
42. Ince C, Albertella L, Liu C, et al. Problematic pornography use and novel patterns of escalating use: A cross-sectional network analysis with two independent samples. *Addict Behav*. 2024;156:108048. https://doi.org/10.1016/j.addbeh.2024.108048
43. Okabe Y, Ito D. Psychometric Properties of the Problematic Pornography Use Scale in a Japanese Sample. *Arch Sex Behav*. 2022;51(2):1221-1235. https://doi.org/10.1007/s10508-021-02141-2
44. Blum AW, Chamberlain SR, Grant JE. Quality of life of young adults with non-paraphilic problematic sexual behaviors: An exploratory study. *Addict Behav Rep*. 2018;8:164-169. https://doi.org/10.1016/j.abrep.2018.10.003
45. Markert C, Storz F, Golder S, et al. On the current psychotherapeutic situation for persons with pornography use disorder in Germany. *J Behav Addict*. 2023;12(2):421-434. https://doi.org/10.1556/2006.2023.00011
46. Koós M, Bőthe B, Orosz G, Potenza MN, Reid RC, Demetrovics Z. The negative consequences of hypersexuality: Revisiting the factor structure of the Hypersexual Behavior Consequences Scale and its correlates in a large, non-clinical sample. *Addict Behav Rep*. 2020;13:100321. https://doi.org/10.1016/j.abrep.2020.100321
47. Reid RC, Garos S, Fong T. Psychometric development of the hypersexual behavior consequences scale. *J Behav Addict*. 2012;1(3):115-122. https://doi.org/10.1556/JBA.1.2012.001
48. Chumakov EM, Petrova NN, Kraus SW. Compulsive sexual behavior in HIV-Infected men in a community based sample, Russia. Sex Health Compuls. 2019;26(1-2):164-175. https://doi.org/10.1080/10720162.2019.1582379
49. Kalichman SC, Cain D. The relationship between indicators of sexual compulsivity and high risk sexual practices among men and women receiving services from a sexually transmitted infection clinic. *J Sex Res*. 2004;41(3):235-241. https://doi.org/10.1080/00224490409552231
50. First MB, Reed GM, Hyman SE, Saxena S. The development of the ICD-11 Clinical Descriptions and Diagnostic Guidelines for Mental and Behavioural Disorders. *World Psychiatry.* 2015;14(1):82-90. https://doi.org/10.1002/wps.20189
51. Griffin KR, Way BM, Kraus SW. Controversies and clinical recommendations for the treatment of Compulsive Sexual behavior Disorder. *Curr Addict Rep*. 2021;8(4):546-555. https://doi.org/10.1007/s40429-021-00393-5
52. Bőthe B, Koós M, Tóth-Király I, Orosz G, Demetrovics Z. Investigating the Associations Of Adult ADHD Symptoms, Hypersexuality, and Problematic Pornography Use Among Men and Women on a Largescale, Non-Clinical Sample. *J Sex Med*. 2019;16(4):489-499. https://doi.org/10.1016/j.jsxm.2019.01.312
53. Grant Weinandy JT, Lee B, Hoagland KC, Grubbs JB, Bőthe B. Anxiety and Compulsive Sexual Behavior Disorder: A Systematic Review. *J Sex Res*. 2023;60(4):545-557. https://doi.org/10.1080/00224499.2022.2066616
54. Kraus SW, Potenza MN, Martino S, Grant JE. Examining the psychometric properties of the Yale-Brown Obsessive-Compulsive Scale in a sample of compulsive pornography users. *Compr Psychiatry*. 2015;59:117-122. https://doi.org/10.1016/j.comppsych.2015.02.007
55. Reid RC, Carpenter BN, Gilliland R, Karim R. Problems of self-concept in a patient sample of hypersexual men with attention-deficit disorder. *J Addict Med*. 2011;5(2):134-140. https://doi.org/10.1097/ADM.0b013e3181e6ad32
56. Rooney BM, Tulloch TG, Blashill AJ. Psychosocial Syndemic Correlates of Sexual Compulsivity Among Men Who Have Sex with Men: A Meta-Analysis. *Arch Sex Behav*. 2018;47(1):75-93. https://doi.org/10.1007/s10508-017-1032-3
57. Schultz K, Hook JN, Davis DE, Penberthy JK, Reid RC. Nonparaphilic hypersexual behavior and depressive symptoms: a meta-analytic review of the literature. *J Sex Marital Ther*. 2014;40(6):477-487. https://doi.org/10.1080/0092623X.2013.772551
58. Soldati L, Bianchi-Demicheli F, Schockaert P, et al. Association of ADHD and hypersexuality and paraphilias. *Psychiatry Res*. 2021;295:113638. https://doi.org/10.1016/j.psychres.2020.113638
59. Lew-Starowicz M, Lewczuk K, Nowakowska I, Kraus S, Gola M. Compulsive Sexual Behavior and Dysregulation of Emotion. *Sex Med Rev*. 2020;8(2):191-205. https://doi.org/10.1016/j.sxmr.2019.10.003
60. Ballester-Arnal R, Castro-Calvo J, Giménez-García C, Gil-Juliá B, Gil-Llario MD. Psychiatric comorbidity in compulsive sexual behavior disorder (CSBD). *Addict Behav*. 2020;107:106384. https://doi.org/10.1016/j.addbeh.2020.106384
61. De Giorgi R, Series H. Treatment of Inappropriate Sexual Behavior in Dementia. *Curr Treat Options Neurol*. 2016;18(9):41. https://doi.org/10.1007/s11940-016-0425-2
62. Kopeykina I, Kim HJ, Khatun T, et al. Hypersexuality and couple relationships in bipolar disorder: A review. *J Affect Disord*. 2016;195:1-14. https://doi.org/10.1016/j.jad.2016.01.035
63. Weintraub D, Claassen DO. Impulse Control and Related Disorders in Parkinson's Disease. *Int Rev Neurobiol*. 2017;133:679-717. https://doi.org/10.1016/bs.irn.2017.04.006
64. Kraus SW, Martino S, Potenza MN. Clinical Characteristics of Men Interested in Seeking Treatment for Use of Pornography. *J Behav Addict*. 2016;5(2):169-178. https://doi.org/10.1556/2006.5.2016.036
65. Kowalewska E, Gola M, Lew-Starowicz M, Kraus SW. Predictors of Compulsive Sexual Behavior Among Treatment-Seeking Women. *Sex Med*. 2022;10(4):100525. https://doi.org/10.1016/j.esxm.2022.100525
66. Bőthe B, Tóth-Király I, Potenza MN, Orosz G, Demetrovics Z. High-Frequency Pornography Use May Not Always Be Problematic. *J Sex Med*. 2020;17(4):793-811. https://doi.org/10.1016/j.jsxm.2020.01.007
67. Jennings TL, Gleason N, Kraus SW. Assessment of compulsive sexual behavior disorder among lesbian, gay, bisexual, transgender, and queer clients•. *J Behav Addict*. 2022;11(2):216-221. https://doi.org/10.1556/2006.2022.00028
68. Jennings TL, Gleason N, Pachankis JE, Bőthe B, Kraus SW. LGBQ-affirming clinical recommendations for compulsive sexual behavior disorder. *J Behav Addict*. 2024;13(2):413-428. https://doi.org/10.1556/2006.2024.00012
69. Maniaci MR, Rogge RD. Caring about carelessness: Participant inattention and its effects on research. *J Res Pers*. 2013;48:61-83. https://doi.org/10.1016/j.jrp.2013.09.008
70. Ministry of Internal Affairs and Communications. Types of local governments. Accessed March 1, 2022. https://www.soumu.go.jp/english/index.html
71. Okabe Y, Ito D. Properties of the Compulsive Sexual Behavior Disorder Scale-19 among Nationally Representative Sample in Japan. *Int J Ment Health Addiction*. 2024;22:3709–3732. https://doi.org/10.1007/s11469-023-01077-z
72. Bőthe B, Bartók R, Tóth-Király I, et al. Hypersexuality, Gender, and Sexual Orientation: A Large-Scale Psychometric Survey Study. *Arch Sex Behav*. 2018;47(8):2265-2276. https://doi.org/10.1007/s10508-018-1201-z.
73. Hald GM, Malamuth NM. Self-perceived effects of pornography consumption. *Arch Sex Behav*. 2008;37(4):614-625. https://doi.org/10.1007/s10508-007-9212-1
74. Reid RC, Li DS, Gilliland R, Stein JA, Fong T. Reliability, validity, and psychometric development of the pornography consumption inventory in a sample of hypersexual men. *J Sex Marital Ther*. 2011;37(5):359-385. https://doi.org/10.1080/0092623X.2011.607047
75. Kor A, Zilcha-Mano S, Fogel YA, Mikulincer M, Reid RC, Potenza MN. Psychometric development of the Problematic Pornography Use Scale. *Addict Behav*. 2014;39(5):861-868. https://doi.org/10.1016/j.addbeh.2014.01.027
76. Development of the World Health Organization WHOQOL-BREF quality of life assessment. The WHOQOL Group. *Psychol Med*. 1998;28(3):551-558. https://doi.org/10.1017/s0033291798006667
77. Norton R. Measuring Marital Quality: A critical look at the dependent variable. *J Marriage Fam*. 1983;45(1):141. https://doi.org/10.2307/351302
78. Moroi K. Perceptions of Equity in the Division of Household Labor. *Jpn J Fam Psychol*.1996;10:15–30. https://doi.org/10.57469/jafp.10.1_15
79. Kimoto Y, Ikeda S, Nagao K, et al. The new Japanese versions of International Index of Erectile function (IIEF) and IIEF5. *Jpn J Impot Res*. 2009;24: 295–308.
80. Rosen RC, Riley A, Wagner G, Osterloh IH, Kirkpatrick J, Mishra A. The international index of erectile function (IIEF): a multidimensional scale for assessment of erectile dysfunction. *Urology*. 1997;49(6):822-830. https://doi.org/10.1016/s0090-4295(97)00238-0
81. Sheehan DV. The anxiety disease. Scribner; 1983.
82. Yoshida T, Otsubo T, Tsuchida H, Wada Y, Kamijima K, Fukui K. Reliability and validity of the Sheehan Disability Scale-Japanese version. *Jpn J Clin Psychopharmacol*. 2004;7:1645–1653.
83. Kroenke K, Spitzer RL, Williams JB. The PHQ-9: validity of a brief depression severity measure. *J Gen Intern Med*. 2001;16(9):606-613. https://doi.org/10.1046/j.1525-1497.2001.016009606.x
84. Muramatsu K, Miyaoka H, Kamijima K, et al. Performance of the Japanese version of the Patient Health Questionnaire-9 (J-PHQ-9) for depression in primary care. *Gen Hosp Psychiatry*. 2018;52:64-69. https://doi.org/10.1016/j.genhosppsych.2018.03.007
85. Ubara A, Okajima I, Machida N, Kadotani H, Ishikawa S. Development of the Japanese version of the Sleep-related Behaviors Questionnaire-Short form: Evaluation of its reliability and validity. *Jpn J Behav Med*. 2020;25(2): 216–226. https://doi.org/10.11331/jjbm.25.216
86. Muramatsu K. An up-to-date letter in the Japanese version of PHQ, PHQ-9, PHQ-15. *Niigata Seiryo Univ Grad Sch Clin Psychol Res*. 2014;7:35–39. https://doi.org/10.32147/00001605
87. Spitzer RL, Kroenke K, Williams JB, Löwe B. A brief measure for assessing generalized anxiety disorder: the GAD-7. *Arch Intern Med*. 2006;166(10):1092-1097. https://doi.org/10.1001/archinte.166.10.1092
88. Muramatsu K, Miyaoka H, Muramatsu Y, et al. Validation and utility of a Japanese version of the GAD-7. *Jpn J Psychosom Med*. 2010;50(6):592. https://doi.org/10.15064/jjpm.50.6_592_2
89. Doi S, Ito M, Takebayashi Y, Muramatsu K, Horikoshi M. Factorial Validity and Invariance of the 7-Item Generalized Anxiety Disorder Scale (GAD-7) Among Populations With and Without Self-Reported Psychiatric Diagnostic Status. *Front Psychol*. 2018;9:1741. https://doi.org/10.3389/fpsyg.2018.01741
90. Kessler RC, Adler L, Ames M, et al. The World Health Organization Adult ADHD Self-Report Scale (ASRS): a short screening scale for use in the general population. *Psychol Med*. 2005;35(2):245-256. https://doi.org/10.1017/s0033291704002892
91. Takeda T, Tsuji Y, Kurita H. Psychometric properties of the Japanese version of the Adult Attention-deficit hyperactivity disorder (ADHD) Self-Report Scale (ASRS-J) and its short scale in accordance with DSM-5 diagnostic criteria. *Res Dev Disabil*. 2017;63:59-66. https://doi.org/10.1016/j.ridd.2017.02.011
92. Wild D, Grove A, Martin M, et al. Principles of Good Practice for the Translation and Cultural Adaptation Process for Patient-Reported Outcomes (PRO) Measures: report of the ISPOR Task Force for Translation and Cultural Adaptation. *Value Health*. 2005;8(2):94-104. https://doi.org/10.1111/j.1524-4733.2005.04054.x
93. Cohen J. *Statistical Power Analysis for the Behavioral Sciences*. 2th ed. Routledge; 1988. https://doi.org/10.4324/9780203771587
94. Cohen J. A power primer. *Psychol Bull*. 1992;112(1):155-159. <https://doi.org/10.1037/0033-2909.112.1.155>
95. JASP Team. JASP (Version 0.18.3) [Computer software]. 2024. https://jasp-stats.org/
96. Harper C, Hodgins DC. Examining Correlates of Problematic Internet Pornography Use Among University Students. *J Behav Addict*. 2016;5(2):179-191. https://doi.org/10.1556/2006.5.2016.022
97. Slavin MN, Blycker GR, Potenza MN, Bőthe B, Demetrovics Z, Kraus SW. Gender-Related Differences in Associations Between Sexual Abuse and Hypersexuality. *J Sex Med*. 2020;17(10):2029-2038. https://doi.org/10.1016/j.jsxm.2020.07.008
98. Vaillancourt-Morel MP, Godbout N, Sabourin S, Briere J, Lussier Y, Runtz M. Adult Sexual Outcomes of Child Sexual Abuse Vary According to Relationship Status. *J Marital Fam Ther*. 2016;42(2):341-356. https://doi.org/10.1111/jmft.12154
99. Takayama, A. Marriage, aging, and women’s pursuit of commercial sex in Japan. *Sexualities*. 2021; 24(4): 592-613. https://doi.org/10.1177/1363460720973909
100. Dwulit AD, Rzymski P. The Potential Associations of Pornography Use with Sexual Dysfunctions: An Integrative Literature Review of Observational Studies. *J Clin Med*. 2019;8(7):914. https://doi.org/10.3390/jcm8070914
101. Bőthe B, Tóth-Király I, Demetrovics Z, Orosz G. The pervasive role of sex mindset: Beliefs about the malleability of sexual life is linked to higher levels of relationship satisfaction and sexual satisfaction and lower levels of problematic pornography use. *Pers Individ Differ*. 2017;117:15-22. https://doi.org/10.1016/j.paid.2017.05.030
102. Bőthe B, Tóth-Király I, Bella N, Potenza MN, Demetrovics Z, Orosz G. Why do people watch pornography? The motivational basis of pornography use. *Psychol Addict Behav.* 2021;35(2):172-186. https://doi.org/10.1037/adb0000603
103. Daspe MÈ, Vaillancourt-Morel MP, Lussier Y, Sabourin S, Ferron A. When Pornography Use Feels Out of Control: The Moderation Effect of Relationship and Sexual Satisfaction. *J Sex Marital Ther*. 2018;44(4):343-353. https://doi.org/10.1080/0092623X.2017.1405301
104. Wright PJ, Tokunaga RS, Kraus A, Klann E. Pornography Consumption and Satisfaction: A Meta-Analysis. *Hum Commun Res*. 2017;43(3):315-343. https://doi.org/10.1111/hcre.12108
105. Kalichman SC, Rompa D. Sexual sensation seeking and Sexual Compulsivity Scales: reliability, validity, and predicting HIV risk behavior. *J Pers Assess*. 1995;65(3):586-601. https://doi.org/10.1207/s15327752jpa6503_16
106. Fernandez DP, Griffiths MD. Psychometric Instruments for Problematic Pornography Use: A Systematic Review. *Eval Health Prof*. 2021;44(2):111-141. https://doi.org/10.1177/0163278719861688
107. Harada T. Compulsive sexual behaviour. *Clinical Psychiatry*. 2019;61(3):277–283. https://doi.org/10.11477/mf.1405205794
108. Miner MH, Romine RS, Raymond N, Janssen E, MacDonald A 3rd, Coleman E. Understanding the Personality and Behavioral Mechanisms Defining Hypersexuality in Men Who Have Sex With Men. *J Sex Med*. 2016;13(9):1323-1331. https://doi.org/10.1016/j.jsxm.2016.06.015
109. Dhuffar MK, Griffiths MD. Barriers to female sex addiction treatment in the UK. *J Behav Addict*. 2016;5(4):562-567. https://doi.org/10.1556/2006.5.2016.072
110. Gola M, Lewczuk K, Potenza MN, et al. What should be included in the criteria for compulsive sexual behavior disorder?. *J Behav Addict*. 2022;11(2): 160-165. https://doi.org/10.1556/2006.2020.00090
